# Allergenic response induced by *Pterobothrium crassicolle* (Cestoda: Trypanorhyncha) extracts in murine model

**DOI:** 10.1590/S1984-29612024039

**Published:** 2024-07-22

**Authors:** Danuza Pinheiro Bastos Garcia de Mattos, Maurício Afonso Verícimo, Sérgio Carmona de São Clemente, Michelle Cristie Gonçalves da Fonseca, Marcelo Knoff

**Affiliations:** 1 Departamento de Microbiologia e Parasitologia, Instituto Biomédico, Universidade Federal Fluminense – UFF, Niterói, RJ, Brasil; 2 Laboratório de Imunobiologia das Doenças Infecciosas e Granulomatosas, Instituto de Biologia, Universidade Federal Fluminense – UFF, Niterói, RJ, Brasil; 3 Laboratório de Inspeção e Tecnologia de Pescado, Departamento de Tecnologia de Alimentos, Faculdade de Veterinária, Universidade Federal Fluminense – UFF, Niterói, RJ, Brasil; 4 Laboratório de Helmintos Parasitos de Vertebrados, Instituto Oswaldo Cruz, Fundação Oswaldo Cruz – Fiocruz, Rio de Janeiro, RJ, Brasil

**Keywords:** IgE, IgG, immunoblot, passive cutaneous anaphylaxis, fish parasite, IgE, IgG, imunoblot, anafilaxia cutânea passiva, parasito de peixe

## Abstract

The aim of this study was to determine the allergenic activity of components present in crude extracts of *Pterobothrium crassicolle* plerocerci (CPE) and blastocysts (CBE) obtained from *Micropogonias furnieri* in a murine model. Two groups of seven animals each received 50 µg of CPE or CBE on days 1, 35 and 120. Serum samples were tested by ELISA and Immunoblotting. Specific IgG and IgE levels were detected by ELISA, showing specific humoral responses for the primary immunization for both immunoglobulins and continuously growing titers for IgE. Positive Passive Cutaneous Anaphylaxis tests in rats sensitized with anti-CBE sera and tested by CBE, showed biologically, the allergenic activity of the extracts. The CPE and CBE showed some different recognition regions but both experimental groups recognized all regions of the extracts when tested for cross reactions, showing that CPE and CBE could share antigenic recognition sites.

## Resumo

O objetivo deste estudo foi determinar a atividade alergênica de componentes presentes em extratos crus de plerocercos (CPE) e de blastocistos de *Pterobothrium crassicolle* (CBE), obtidos de *Micropogonias furnieri*, em modelo murino. Dois grupos de sete animais receberam cada um 50 µg de CPE ou CBE nos dias 1, 35 e 120. As amostras de soro foram testadas por ELISA e Imunoblot. Os níveis específicos de IgG e IgE foram detectados por ELISA, mostrando respostas humorais específicas para a imunização primária para ambas as imunoglobulinas e títulos crescentes de IgE. Testes positivos de Anafilaxia Cutânea Passiva em ratos sensibilizados com soros anti-CBE e testados por CBE, demonstraram biologicamente, a atividade alergênica dos extratos. O CPE e o CBE evidenciaram algumas regiões de reconhecimento diferentes, mas ambos os grupos experimentais reconheceram todas as regiões dos extratos, quando testados para reações cruzadas, mostrando que o CPE e o CBE poderiam compartilhar locais de reconhecimento antigênico.

## Introduction

Trypanorhynch cestodes are distributed worldwide, especially in tropical and subtropical regions. They are amongst the most common metazoan parasites of marine fish (Palm, 2004), although freshwater organisms have also been reported as hosts (Rego, 1982, 1987; Campbell et al., 1999). Adult cestodes are found in the stomach and intestine of elasmobrachs and metacestodes parasitize a wide variety of invertebrates and teleost fish (Campbell & Beveridge, 1994; Palm et al., 2009). Their presence in the body cavity, mesenteries, viscera serosa, and especially in the flesh of teleost fish may compromise the commercial value of the stock, causing significant economic losses (Palm, 1997; Palm et al., 1993, 1994; Petersen et al., 1993; Al-Zubaidy & Mhaisen, 2011; Dias et al., 2011; Fonseca et al., 2012; Zuchinalli et al., 2016; Oliveira et al., 2019; Diniz et al., 2022; Leite et al., 2022; Miguel et al., 2022; Menezes et al., 2023).

Species belonging to the *Pterobothrium* Diesing, 1850 (Pterobothriidae Pintner, 1931) genus have been reported parasitizing various teleost fish species, including their flesh, in Australia, Indonesia, Sri Lanka, India, Persian Gulf, West African coast, Gulf of Mexico and the Atlantic coast of South America (Diesing, 1850; Rego et al., 1974; Overstreet, 1977; Rego, 1987; São Clemente et al., 1991; Palm et al., 1994; 2009; Campbell & Beveridge, 1996; Palm, 1997; Moore et al., 2003, 2011; Zischke et al., 2009; Charters et al., 2010; Felizardo et al., 2010; Haseli et al., 2010, 2011; Dias et al*.*, 2011; Fonseca et al., 2012; Schaeffner & Beveridge, 2012; Eiras et al., 2016; Zuchinalli et al., 2016; Beveridge et al., 2017; Oliveira et al., 2019; Diniz et al., 2022; Leite et al., 2022; Miguel et al., 2022; Menezes et al., 2023).

*Micropogonias furnieri* (Desmarest, 1823), known as the whitemouth croaker, is an important commercially exploited marine fish which inhabits the Atlantic Ocean from the Gulf of Mexico to Argentina and is frequently parasitized by trypanhorhynch, especially *Pterobothrium* species (Diesing, 1850; Rego et al., 1974; Overstreet, 1977, 1978; São Clemente, 1986a, b, 1987; Rego, 1987; Pereira, 1993; Alves & Luque, 2001; Pereira & Boeger, 2005; Porto et al., 2009; Eiras et al., 2016).

Due to the increasing worldwide consumption of raw, undercooked or poorly processed fish, human accidental infections with fish parasites and some allergic related reactions have represented a serious public health hazard, with increasing medical concern in several countries (Chai et al., 2005; Audicana & Kennedy, 2008; Dorny et al., 2009; Broglia & Kapel, 2011). Human parasitism by trypanorhynch cestodes is extremely rare (Kikuchi et al., 1981; Fripp & Mason, 1983), however Pelayo et al. (2009) showed the seroprevalence of an immune response against the trypanorhynch *Gymnorhynchus gigas* in a Spanish population. According to Deardorff et al. (1984), the metacestode toxins are gradually released to the fish tissues, mostly flesh, which could represent a hazard for human health, and experimental studies have highlighted the risk of allergic reactions by trypanorhynchs (Rodero & Cuéllar, 1999; Vázquez-López et al., 2001, 2002; Gòmez-Morales et al., 2008; Mattos et al., 2015).

Considering the lack of data about the allergenic potential of Pterobothriidae trypanorhynchs, the aim of the present study was to determine if crude extracts of *Pterobothrium crassicolle* (Diesing, 1850) plerocercoids and blastocysts have antigenic compounds able to induce specific allergic responses in experimental murine model.

## Material and Methods

A total of 107 specimens of *M*. *furnieri* (24.0-65.0 cm) were obtained from fish markets and fishermen in the municipalities of Niterói and Cabo Frio, Rio de Janeiro State, Brazil, between March/2009 and March/2012. They were collected and transported on ice in isothermic bags for examination at the Laboratório de Inspeção e Tecnologia de Pescado, Faculdade de Veterinária (Fish Inspection and Technology Laboratory, Faculty of Veterinary), Universidade Federal Fluminense (UFF). The fish specimens were identified according to Menezes & Figueiredo (1980) and submitted to necropsy at the laboratory. Parasite recovery was carried out according to the methodology proposed by Eiras et al. (2006). The taxonomic identification of trypanorhynch cestodes was based on Campbell & Beveridge (1996) and identified as *P. crassicolle* metacestode. The plerocerci of *P. crassicolle* and its blastocysts were manually collected from the fish with the aid of scissors and forceps.

The metacestodes were transported on ice inside isothermic bags to the Laboratório de Imunobiologia das Doenças Infecciosas e Granulomatosas, Departamento de Imunologia, Instituto de Biologia (Department of Immunobiology, Institute of Biology), UFF, where immunological analyses were carried out. The crude plerocerci extract (CPE) and the crude blastocysts extract (CBE) were obtained after separation of the metacestode parts in different containers, followed by extensive washing using sterile 0.1M phosphate-buffered saline (PBS), pH 7.3, supplemented with 5% penicillin and 5% streptomycin. The metacestode parts were homogenized singly in a Potter-Elvehjem homogenizer (Thomas Scientific, PA, USA) after a final wash with non-supplemented, sterile PBS. The homogenate was then submitted to six 30-s cycles using the Tissue Ruptor (Qiagen Instruments AG, Zurich, Switzerland), the suspension obtained centrifuged at 60.000 g at 4ºC for 30 minutes and the supernatant filtered through a 0.22 µm MillexGV Millipore filter (Millipore, France).

The same protocol was used to prepare the crude fish protein extract (CFE) of *M. furnieri*, which was used as the control antigen for the serological assays. The protein contents of the CPE, CBE and CFE were estimated according to Lowry et al. (1951).

To determine the molecular weight range of the CPE, 0.03mg of the extract was submitted to SDS-PAGE (sodium dodecyl sulphate-polyacrylamide gel electrophoresis) using a 12%, 100 x 100mm gel (Vertical System, Bio-Rad, Hercules, California, USA) for 2h at 140V (Laemmli, 1970).

Ten-week-old female BALB/c mice were maintained in separate cages according to their experimental group (two experimental groups [n=7] and control group [n=5]), receiving distilled water and food (Nuvilab CR-1, Nuvital Nutrientes S/A, Brazil) *ad libitum*. All animals were injected with xylazine (200 μg/kg g/kg body weight) intramuscularly associated with ketamine (10 mg/kg body weight) before invasive procedures. Euthanasia was applied using an overdose of anesthetic drugs. The study was approved by the Animal Research Ethics Committee of the UFF Centre for Laboratory Animals (038∕2009).

Each experimental group was immunized intraperitonally (i.p.) on days 1, 35 and 120, with a suspension containing 50 µg of CPE or CBE and 2.0 mg of commercial aluminum hydroxide solution, Al(OH)_3,_ in a final volume corresponding to 200 μl of suspension. At the same times the control group was injected with a suspension containing only sterile saline and aluminum hydroxide.

Blood samples were collected from each animal from the retro-orbital plexus (pre-immunization for paired controls) after 14, 21, 35, 42, 49, 56, 120, 127 and 135 days (post-immunization). The samples were centrifuged to obtain three sera, which were stored at -20ºC until examined.

The specific IgG and IgE levels in the CPE and CBE were determined using an enzyme-linked immunosorbent assay (ELISA) (Antunes et al., 2009). Briefly, 96-well microtiter plates (Nunc-Imuno^TM^ Plate Maxi Sorp^TM^ surface; Nalge Nunc International, Rochester, New York, USA) were coated with 20 µg/ml (1 µg/well) of CPE or CBE or CFE eluted in 0.1M carbonate buffer, pH 9.6, overnight. The sera were serially diluted and incubated for 2 h at 37ºC for the detection of IgG (1:100, 1:300, 1:900 and 1:2700 in PBS v/v) and IgE (1:100; 1:200; 1:400 and 1:800). The peroxidase-conjugated antibodies: anti-total IgG (L and H) (1:10,000) (rabbit anti-mouse IgG, whole molecule, Sigma-Aldrich, St. Louis, Missouri, USA) and anti-IgE (Σchain) (rat anti-mouse IgE, Invitrogen, Carlsbad, California, USA) antibodies (50 mL/well) were used, as recommended by the manufacturers. Reactions were developed using 50 µL/well of OPD substrate (0.04% *O*-phenylene-diamine [Sigma-Aldrich] plus 0.04% hydrogen peroxide in a phosphate-citrate buffer [pH 5.0]). The chromogenic reaction was stopped with 50 µL/well of 3N sulfuric acid. The optical density (OD) was determined by spectrophotometry (Anthos 2010, Krefeld, Germany) at 492 nm. The ELISA scores were computed by summing up the ODs between 1:100 and 1:2700 (IgG) or between 1:100 and 1:800 (IgE) of the serum dilutions (an approximate calculation of the area under the dilution curve). Each score represents the mean ± standard error of the mean (SEM) for each experimental group. Cross reactivity to fish proteins was assessed using an IgG ELISA essentially as described above, using MF-CPE as the antigen, and following the same protocol.

Six female Lou-M adult rats each weighing 150g were reared in the animal house of the UFF and tested using the Passive Cutaneous Anaphylaxis (PCA) assay. This technique, as described by Braga & Mota (1976), uses a 72 h sensitization period for the IgE antibody. Briefly, a shaved dorsal area was injected intradermally with 30 μL of mice sera from the CPE, CBE or control groups (days 56, 120, 127 and 135) diluted 1:40. After the sensitization period, PCA reactions attributable to the IgE class were elucidated by the rats by the intravenous administration of 500 μg of CPE, CBE or CFE in 0.5 mL saline mixed with 1% Evans blue dye. Saline (0.5 mL) was used as the negative control. Thirty minutes later, the rats were euthanized by an overdose of anesthetic drugs. The dorsal skin was removed and inverted to observe and measure any pigmented area and the reactions considered positive for spots larger than 5 mm in diameter.

The recognition of immunogenic proteins by Immunoblotting (Western Blot) was used to determine the reactivity profile of specific IgG and IgE. For the western blot, 0.3 mg of CPE and CBE were submitted to the same SDS-PAGE conditions, followed by transfer of the protein bands from the separating gel to the nitrocellulose membrane using a Semi-dry blotter (Bio-Rad, CA, USA). Subsequently, the membranes were blocked overnight with 5% fat-free milk (Nestle) in PBS solution, washed with 0.05% PBS-Tween, dried at room temperature (RT) and cut into strips. Two strips were incubated overnight at RT with each serum sample diluted 1:100 v/v in blocking buffer, with constant rocking. After washing four times with TBS- (Tris-buffered saline) Tween, one membrane strip of each serum was incubated with peroxidase-labelled goat anti-mouse IgG (Bio-Rad) for 2h and the other exposed to rat anti-mouse IgE (Invitrogen) for 3h, followed by HRP-goat anti-rat IgG (H + L, Bio-Rad, CA, USA) for 2h at RT with constant rocking. After at final wash, the peroxidase substrate (3.3’-diaminobenzidine, Sigma-Aldrich, USA) was added to develop the Ag/IgG or Ag/IgE interaction. All antibodies were used according to the manufacturer’s recommendations.

The Shapiro-Wilk test was used to assess normality. Data were evaluated using the General Linear Model, with repeated measures ANOVA and Bonferroni post-hoc. The software used was SPSS (IBM, version 24). In the statistical analysis of experimental data, the values were considered significant at p< 0.05.

## Results

**Figure 1:**
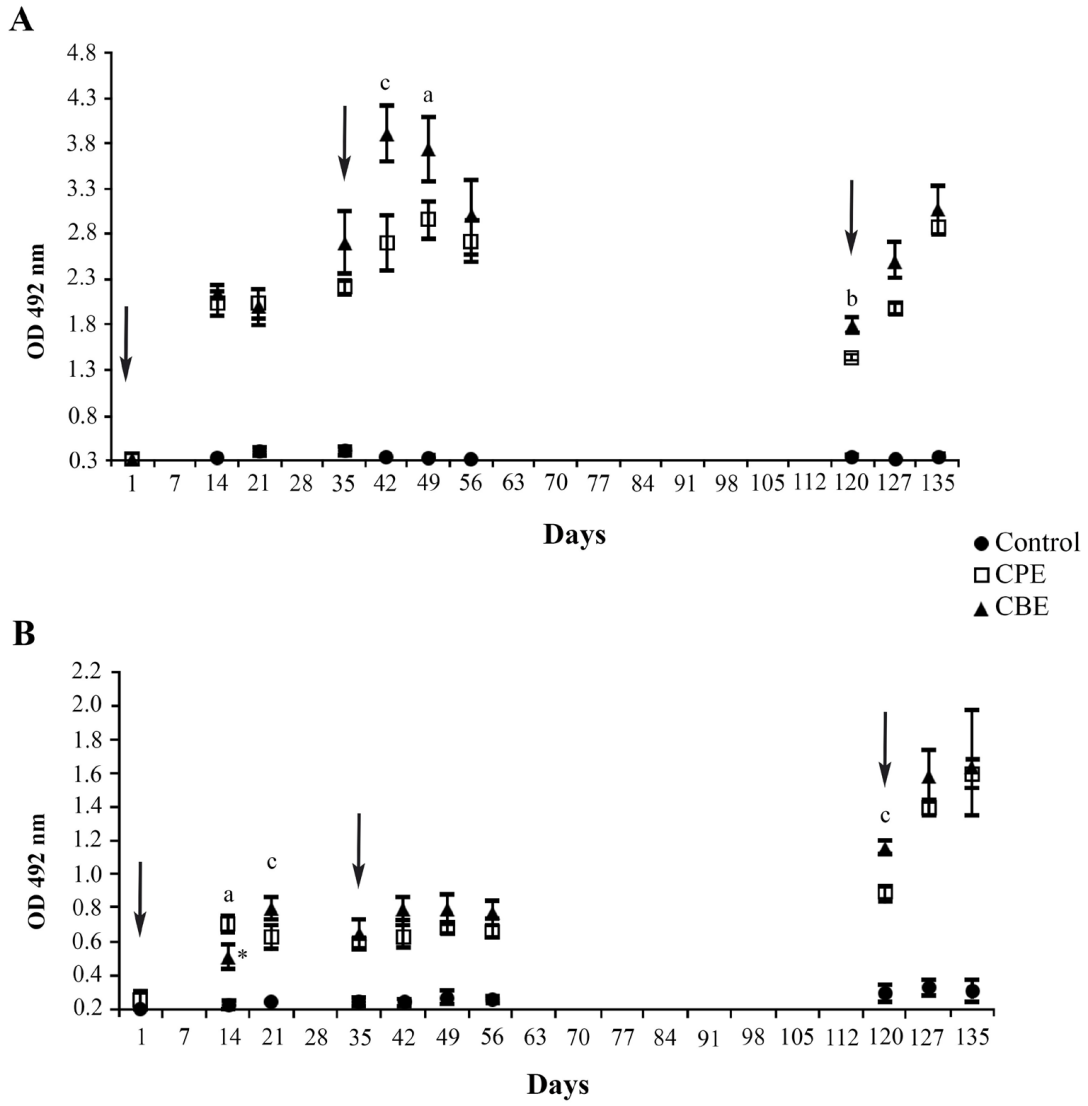
Dynamics of the specific IgG (A) and IgE (B) serum levels. Two groups, each with 7 mice, received intraperitoneally 50 µg of crude extract of *Pterobothrium crassicolle* plerocerci - CPE (square) or blastocysts - CBE (triangle) associated with 2 mg Al(OH)_3_, on days 0, 35 and 120 (arrow). A control group (circle) with 5 animals, received saline solution with 2 mg Al(OH)_3_ on the same days using the same pathway. The values indicate the means of the sums of the optical densities (OD). +/- standard error of the mean of each group. As from day 14, the IgG and IgE levels of both experimental groups were p<0.001 (exception *p<0.01) when compared to the control. ^a^ p<0.05, ^b^ p<0.01, ^c^ p<0.001 between groups.

After the primary immunization, specific IgG and IgE were detected in the serum samples of the experimental groups as from day 14, with statistically significant increasing levels (p<0.001 for all, except for IgE of the CBE group, which was p<0.01 at day 14) when compared with the control group sera. The highest IgG level was observed for the samples collected on day 42 from animals immunized with 50µg of CBE. The titers of specific IgE increased continuously for both the CPE and CBE groups during the experimental period (Figure 1).

Cross-reactions between the immunized groups (CPE and CBE) versus the CFE antigens were not observed by the ELISA assay, and no specific humoral response was detectable in the serum of the animals before the prime immunization or in the control group. However, the serum samples of both experimental groups showed statistically the same recognition of both the parasite extracts.

**Figure 2:**
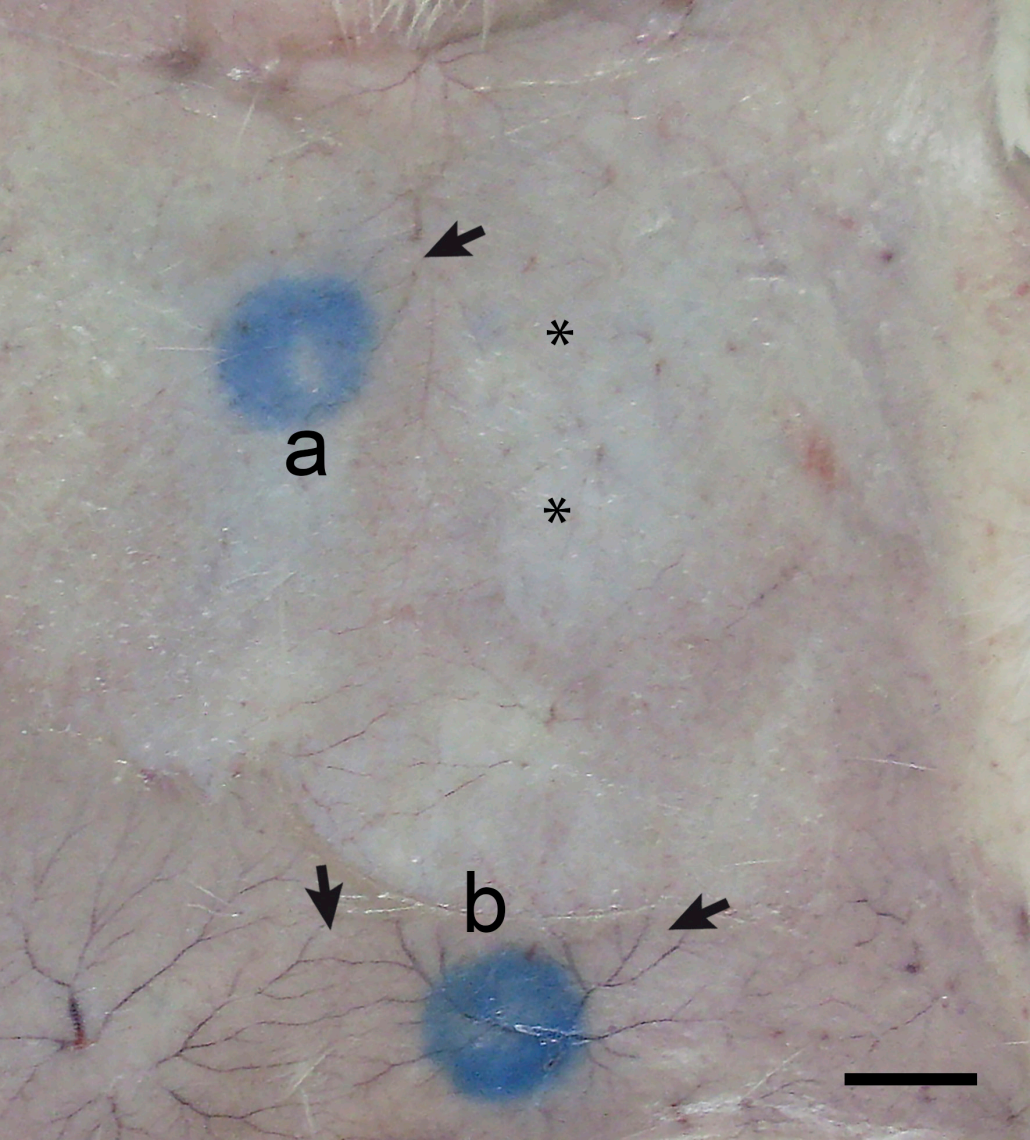
The passive cutaneous anaphylaxis assay (PCA). PCA reaction using Lou-H rat as the receptor of anti-CBE sera from BALB/c mice. Positive PCA reactions for mice sera after 127 (a) and 135 (b) days induced by the CBE. Increased blood influx (arrows); Sera without reaction (asterisks); Crude extract of *Pterobothrium crassicolle* blastocysts (CBE). Bar = 10 mm.

The evaluation of the allergenic properties by Passive Cutaneous Anaphylaxis (PCA) assay allowed for the visualization of profound localized allergic reactions triggered by allergen-induced cross-linking of the FcRI by the binding of allergen-specific IgE located just beneath the skin. The extravasation of Evans blue dye reflected the increase in local vascular permeability, a process that depends on the release of histamine and serotonin mast cell degranulation. The PCA tests were tested by CBE in rats sensitized with anti-CBE serum to indicate the allergenic property of this parasite extract (Figure 2). No reactions were observed in the control serum area or in the CFE tested rat.

**Figure 3:**
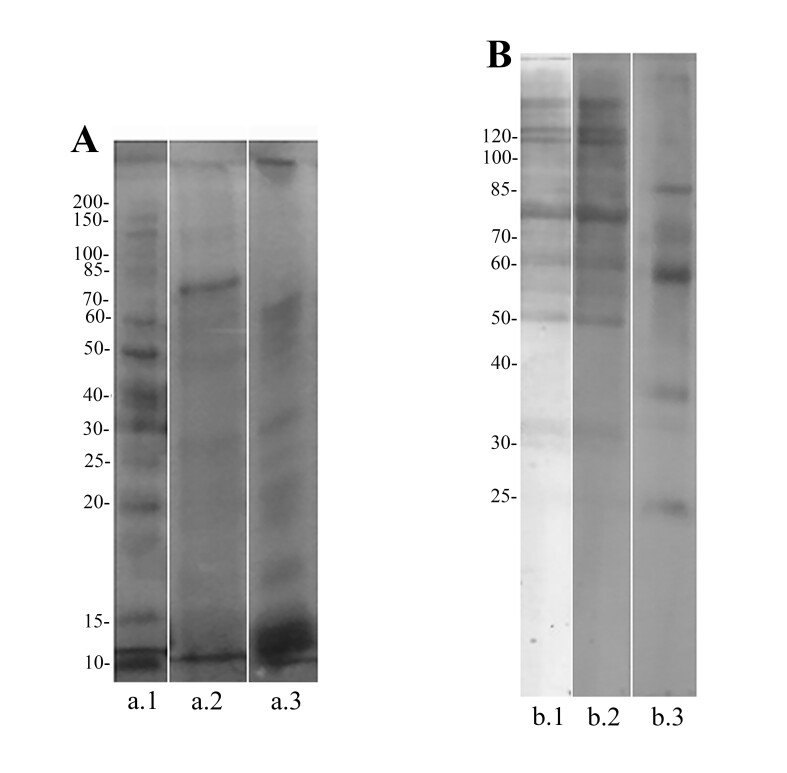
Recognition of immunogenic proteins by immunoblotting. (A) SDS-PAGE (12%) staining by Coomassie brilliant blue showing: the molecular weight (kDa) (a.1), protein profile of CPE (a.2) and CBE (a.3). (B) Immunoblot showing IgG recognition of the immunogenic proteins of CPE (b.1, b.2) and CBE (b.3) in pooled sera from all sensitized mice, seven days after the second immunization (42^nd^ experimental day). Crude extract of *Pterobothrium crassicolle* plerocerci (CPE) and blastocysts (CBE).

In the recognition of immunogenic proteins by immunoblotting most bands were observed between 80 and 15 kDa (CPE) or 70 and 10 kDa (CBE) in the SDS-PAGE. The sharpest CPE band was near to 80 kDa. However, specific IgG recognized CPE proteins with 120 kDa or more, near to 80kDa (sharpest band), 60 kDa, 50 kDa, near 32k, 30 kDa and 25 kDa (Figure 3). However, specific IgG different recognized CBE bands from 120 kDa to 24 kDa with the sharpest bands at 85 kDa, near to 57 kDa, 35kDa and 24kDa. No reactivity was observed in the control serum.

## Discussion and Conclusion

Allergic manifestations to fish parasite antigens are well known and frequently related to the Anisakidae family species, such as *Anisakis simplex* (Audicana & Kennedy, 2008). So far, only a few investigations have been developed to study the allergic potential of other fish parasites such as trypanorhynch cestodes (*Gymnorhynchus gigas* and *Molicola horridus*), using different immunization protocols and administration pathways (Rodero & Cuéllar, 1999; Vázquez-López et al., 2001, 2002; Gòmez-Morales et al., 2008).

The immunogenic capacities of the CPE and CBE after the first, second and third i.p. inoculations were shown by ELISA, with detectable high levels of specific IgG and continuously increasing IgE up to the end of the experiment. Our results corroborate previous data that indicate the use of the murine model with BALB/c mice, testing by i.p. antigenic administration, as appropriate for identifying and characterizing allergens with a protein nature (Rodero & Cuéllar, 1999; Dearman & Kimber, 2001; Vázquez-López et al., 2001; Martínez de Velasco et al., 2002; Gòmez-Morales et al., 2008; van der Ventel et al., 2011).

The cross-reactions observed by ELISA for the CPE and CBE antigens suggest that the two extracts share antigenic recognition sites. Previous studies had discarded the blastocysts and used only the plerocerci, but natural exposure may involve both portions of the metacestodes. The difference between the CPE and CBE antigens with respect to the responses induced was only statistically significant on days 14, 21 (IgE), 42, 49 (IgG) and 120 (IgG and IgE) after the first immunization. In general, CBE induced higher titers of IgG and IgE, but all immunized groups were statistically extremely different from the control group as from 14 days after the first immunization (p<0.001) for both immunoglobulins.

The ELISA and PCA results indicated the allergenic nature of CPE and CBE, since high IgE and IgG (mainly IgG1) levels are known to be related to the regulation of hypersensitivity reactions (Rodero & Cuéllar, 1999; Vázquez-López et al., 2001; Martínez de Velasco et al., 2002).

The SDS-PAGE and Western blot profile of *P. crassicolle* showed similar aspects when compared with other Trypanorhyncha cestodes such as *G. gigas* and *M. horridus*, which also presented IgG binding proteins with similar weight. Vázquez-López et al. (2002) observed a 24 kDa collagenase of *G. gigas* which as recognized by the humoral response of the experimental animals, and Gòmez-Morales et al. (2008) reported IgG binding proteins from *M. horridus* with 26 and 75 kDa. These proteins could be closely related to the IgG binding proteins of *P. crassicolle*.

Since our results indicated the allergenic activity of *P. crassicolle* antigens in murine models, complementary clinical trials are required to elucidate their implications with respect to human health.
